# The Compensatory Tillering in the Forage Grass *Hordeum brevisubulatum* After Simulated Grazing of Different Severity

**DOI:** 10.3389/fpls.2020.00792

**Published:** 2020-06-12

**Authors:** Jihong Yuan, Haiyan Li, Yunfei Yang

**Affiliations:** ^1^Key Laboratory of Vegetation Ecology, Ministry of Education, Institute of Grassland Science, Northeast Normal University, Changchun, China; ^2^Jiangxi Academy of Forestry, Nanchang, China

**Keywords:** vegetative reproduction, compensatory index, grazing tolerance, jointing stage, flowering stage

## Abstract

The response of compensatory growth is an important adaptive strategy for plants to grazing. However, most previous studies on compensatory growth of plants focused on the compensation of the biomass or the number of sexual reproductive offspring and neglected the compensatory growth of vegetative reproduction (VR). This is important not only for plant compensatory growth studies, but also for theoretical and practical studies of grassland production. The clonal tussock grass *Hordeum brevisubulatum* was selected as the research object. Four different clipping severities (unclipping and clipping stubble at heights of 15, 10, and 5 cm) at the jointing stage and flowering stage were implemented to study the effect of simulated grazing. To explore the effect of recovery growth time on plant growth after simulated grazing, three sampling times were used at different recovery times after simulated grazing (1, 3, and 7 weeks). We found that light and moderate grazing severity significantly increased the number of vegetative reproduction modules, the promotion of simulated grazing on the number of vegetative reproduction modules was higher in the jointing stage than the flowering stage, and the increase in simulated grazing severity decreased with prolonged recovery growth time. The number of tillers significantly decreased with the increase in simulated grazing in both the jointing and flowering stages at 1 week after damage, and the decreasing effect weakened with the prolonged recovery growth time. The bud number mainly showed over-compensation, the juvenile tiller number showed complete compensation, and the tiller number showed under-compensation at 1 and 3 weeks after recovery growth. The number of tillers showed complete compensation under different grazing severities in the jointing stage, while it showed under-compensation in the flowering stage at 7 weeks after recovery growth. Our results indicated that different grazing severities in the jointing stage could promote the output of tillers with matter production capacity from vegetative reproduction modules, as well as improve the capability of compensatory growth. Therefore, in plant production, there will be a sustainable development effect on the renewal and productivity of the *H. brevisubulatum* population, resulting in different grazing severities in the jointing stage.

## Introduction

Plant organs are often damaged by grazing in grassland ecosystems at different stages of plant growth. Generally, during the evolution of plant-herbivore interactions, plants adopt compensatory growth strategies to adapt to grazing (Gedge and Maun, 1992). Compensatory growth is defined as the ability of plants to offset the adverse effects of tissue damage, restore organic functionality, and maintain normal growth after grazing (McNaughton, 1983). There are three response patterns of plants to herbivory: harmful, neutral, beneficial, also known as undercompensation; complete compensation; and overcompensation (Belsky, 1986; Georgiadis et al., 1989; Milchunas and Lauenroth, 1993). Plant compensatory growth capacity varies among plant species (Hanley and May, 2006; Tozer et al., 2017) and life forms (Anderson and Frank, 2003; Massad, 2013). For the same species, plant compensatory growth capacity is related not only to the growth status when being grazed (Boege et al., 2007; Tito et al., 2016) but also to the grazing severity (Qian et al., 2017; Quijano-Medina et al., 2019), grazing time (Tucker and Avila-Sakar, 2010; Kornelsen and Avila-Sakar, 2015), and recovery time after grazing (Hendrickson and Briske, 1997). Compensatory growth can be evaluated by a variety of quantitative indicators, such as biomass and number. However, previous studies on compensatory growth have mainly focused on biomass (Erbilgin et al., 2014; Scholes et al., 2016; Wan et al., 2016), while the compensatory growth of countable traits only focused on the number of sexually propagated offspring (Levine and Paige, 2004; Mundim et al., 2012). Little attention has been paid to the compensation of the number of vegetatively propagated offspring in previous studies on plant compensatory growth. Especially for perennial grasses, the importance of vegetative reproduction (VR) has been neglected.

The aboveground tiller population is generally maintained by sexual reproduction through seeds and VR through tillers after perennial grasses are grazed (Coffin and Lauenroth, 1989; Glenn-Lewin et al., 1990; Ott and Hartnett, 2011). Clonal plants with strong VR occupy a large proportion of natural grassland communities dominated by perennial grasses. Therefore, the maintenance and regeneration of community structure mainly depend on VR, and the recruitment from seeds is extremely rare; >99% of tiller regeneration comes from VR (Thompson and Grime, 1979; Benson and Hartnett, 2006; Zhang et al., 2009). The breaking of apical dominance when clonal plants are grazed, especially by large herbivores at different stages of plant growth, promotes the vegetative regeneration or resprouting after damage. The vegetative regeneration of clonal grasses depends on the bud bank, which occurs predominantly from a belowground population of meristems (Ott and Hartnett, 2012; Deng et al., 2015). Resprouting from bud banks is one of the major mechanisms for plant compensatory growth after being grazed (Tuomi et al., 1994; Tiffin, 2000). Previous studies showed that the grazing severities, species identities, growing environment, and sampling time after grazing could affect the dynamics of bud bank input and output (N’Guessan and Hartnett, 2011; Ott et al., 2019; Vidaller et al., 2019). Most studies indicated that grazing would reduce the bud number due to promoting the production of tiller from bud (Busso et al., 1989; Pelaez et al., 2009; N’Guessan and Hartnett, 2011; Ott et al., 2017, 2019). However, the tiller number may increase (Maschinski and Whitham, 1989; Bergelson et al., 1996; Vidaller et al., 2019) or decrease (Busso et al., 1989; N’Guessan and Hartnett, 2011). The tillering response to grazing is one of the major mechanisms conferring plant resilience to herbivory, especially for clonal plants.

During the growth of clonal plants, the sprouting of bud to the formation of tillers constitute the main modules at different stages of VR. In general, these modules consist of bud, juvenile tiller, and tiller at different stages of life history (Yang and Zhu, 2011). The life history pattern of VR, also called the life history stage spectrum, is the relative percentage of bud, juvenile tiller and tiller among the number of total VR modules. Grazing affects the life history pattern by promoting the output of bud and the transformation of VR modules (Ott and Hartnett, 2015a). The transformation of VR and the change in life history patterns are important for grassland ecosystem productivity (Li et al., 2012). Therefore, research on compensatory VR and the structure of VR modules of clonal plants under simulated grazing is not only important for investigating plant compensatory growth but also is of great theoretical and practical significance.

*Hordeum brevisubulatum* is considered a quality forage grass that is widely distributed throughout the grasslands of northeastern China; it is a perennial grass with short rhizomes (Li and Zhang, 1997). To guide pasture management, this study was conducted with different clipping severities at different times to simulate grazing, and different recovery times after damage were also considered. The objective of this study was to explore the effects of simulated grazing severity, grazing time, and recovery time after grazing on compensatory VR and the change in the number and composition of VR modules (tillers, juvenile tillers, and buds). We mainly addressed the following questions: (1) How VR is affected by severity of damage and ontogenetical stage of tillers at the time of damage. (2) How results of VR after damage change with time. (3) How damage affects composition ontogenetical stages of VR modules. (4) How ontogeny and severity of damage affect compensation by VR in *H. brevisubulatum*?

## Materials and Methods

### Experimental Site

The study was conducted at the Grassland Ecological Research Station of the Institute of Grassland Science, which was located in the southern Songnen Grassland of Changling County, Jilin Province (45°45′ N, 123°45′ E). The annual average temperature of this area is 4.9°C, and the annual rainfall and evaporation are 470.6 and 1668 mm, respectively. The experimental site is in a semiarid area with a continental monsoonal climate. The growing season with a frost-free period is 150 days (Yuan et al., 2019). The soil type of the experimental site is aeolian sandy soil.

### Plant Species

*H. brevisubulatum* is a perennial grass species with short rhizomes that is widely distributed throughout the grasslands of northeastern China (Li and Zhang, 1997). This grass species has a strong VR capacity, an early growth and flowering period, long periods of vegetative growth after fruiting, and a late period of senescence. It also has ecological characteristics of drought and salt tolerance. It is considered to be a quality forage grass with a high feeding value due to its high yield and good palatability. The growth period of *H. brevisubulatum* is earlier than that of other species, while the period of senescence is later. *H. brevisubulatum* normally turns green in early April, joints in mid-May, flowers at the end of June, undergoes seed maturation in late June and turns yellow in early October (Wang et al., 2006; Yuan et al., 2019).

### Experimental Design

Seeds of *H. brevisubulatum* were collected from the natural grassland at the field station in 2015 and stored in dry rooms. The nursery garden (2 m × 3 m) was established on July 1, 2016, in an outdoor experiment field, watered regularly to keep the soil moist before the emergence of *H. brevisubulatum*, and artificially weeded regularly to ensure the normal growth of seedlings after the emergence of *H. brevisubulatum*. Six transplanting experimental plots were established on May 1, 2017. The area of each plot (2 m × 3 m) was 6 m^2^. Single seedlings were transplanted with rows 0.3 m apart with 0.3 m between plants. There were 70 tufts in each plot. The plots were irrigated to keep the soil moist after transplanting until all the seedlings survived. Then, the plots were only weeded regularly, without any management of fertilization, irrigation, and insect prevention. The total N content, organic C content and total P of plot soil in the 20-cm-thick soil layer were 1.04 ± 0.04 g kg^–1^, 5.68 ± 0.09 g kg^–1^, and 0.76 ± 0.01 g kg^–1^, respectively. The pH was 8.34 ± 0.02, and the electrical conductivity was 74.41 ± 0.41 mS cm^–1^.

Similarly, sized tufts (10 cm) of *H. brevisubulatum* were chosen for the application of four simulated grazing severities and two simulated grazing stages in 2018. The grazing severities were set as control (unclipping), light (clipping with a stubble height of 15 cm), medium (clipping with a stubble height of 10 cm), and heavy (clipping with a stubble height of 5 cm). 18 tufts (3 tufts in each plot) were treated for each grazing severity conducted in the jointing stage (May 16) and flowering stage (May 31). A total of 144 tufts were treated. All treatment tufts were sampled three times after treatment at each stage, namely, at 1, 3, and 7 weeks after treatment. One treated tuft in each plot for each severity condition at every growth stage was sampled. There were six plots for each condition.

### Harvest and Measurement

There were six sampling times for the jointing and flowering stages. The sampling was conducted by digging out the whole tuft. The entire tuft was dug out in May 24 (1 week after treatment), June 7 (3 weeks after treatment), and July 5 (7 weeks after treatment) for the tufts treated at the jointing stage and removed on June 7 (1 week after treatment), June 21 (3 weeks after treatment), and July 19 (7 weeks after treatment) for the tufts treated at the flowering stage. The aboveground tiller was divided into the tiller and juvenile tiller. The tiller was the old tiller, including the reproductive tiller or vegetative tiller with jointing (the height of the tiller was generally greater than 5 cm). The juvenile tiller was defined as small ramets without jointing, and the height was lower than 5 cm. The numbers of tillers, juvenile tillers, and buds (the length of buds was generally greater than 0.2 cm) at the tiller nodes per tussock were counted.

### Data Analysis

The tiller, juvenile tiller, and bud produced by VR were considered together, and the ratio (R) was calculated.

$${\text{R}}_{\text{i}}=\frac{{\text{N}}_{\text{i}}}{{\text{N}}_{\text{T}}}\times 100$$

R_i_ and N_i_ are the ratio and number of a component of VR modules, respectively, i is a component of VR modules (e.g., bud, juvenile tiller, and tiller); and N_T_ is the total number of the three VR modules (the sum of buds, juvenile tillers and tillers).

The CI based on the number of VR modules was calculated (Tiffin, 2000).

$${\text{CI}}_{\text{i}}={\text{N}}_{\text{it}}\,/\,{\text{N}}_{\text{ic}}$$

CI_i_ is the CI of a component of VR modules, and i is a component of VR modules (e.g., bud, juvenile tiller, tiller). N_it_ and N_ic_ are the numbers of a component of VR modules under the simulated grazing treatment and control, respectively. A value of CI greater than 1 indicates overcompensatory growth, values equal to 1 indicate complete compensatory growth, and values less than 1 indicate undercompensatory growth.

Data were transformed when necessary to achieve normality and homogeneity of variance. Two-way ANOVA analyses were performed to analyze the main and interactive effects of severity of disturbance and ontogeny on the VR capacity and the number, composition, and compensation index of VR modules. The least significant difference (LSD) method was used to make multiple comparisons with the above indexes at different grazing severities. The differences between the jointing stage and flowering stage were analyzed by an independent-sample *t*-test. The differences between the CI and 1 were compared with the use of a single-sample *t*-test. All statistical analyses were performed using SPSS 20.0 statistical software (SPSS Inc., Chicago, IL, United States).

## Results

### The Number of Vegetative Reproduction Modules

**FIGURE 1:**
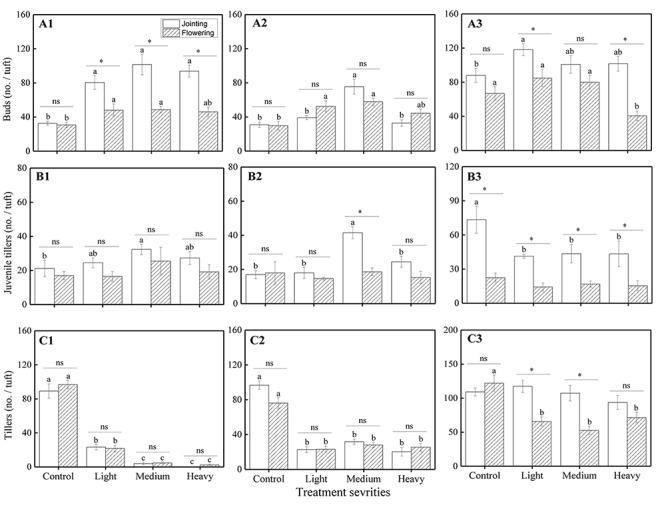
Comparison of the number of VR modules among different grazing severities and at different growing stages of *H. brevisubulatum* tufts at three periods of recovery growth. Control, light, medium, and heavy represent unclipping, clipping with a stubble height of 15 cm, clipping with a stubble height of 10 cm and clipping with a stubble height of 5 cm, respectively. **A–C** represent the number of buds, juvenile tillers and tillers per tuft, respectively. **A1–A3** represent the number of buds at 1, 3 and 7 weeks after recovery growth, respectively. **B1–B3** represent the number of juvenile tillers at 1, 3 and 7 weeks after recovery growth, respectively. **C1–C3** represent the number of tillers at 1, 3 and 7 weeks after recovery growth, respectively. Different lowercase letters represent significant differences among different clipping severities (*p* ≤ 0.05). * and ns indicate that there was a significant difference (*P* ≤ 0.05) and no significant difference, respectively, between the jointing stage and flowering stage.

Statistical results showed that there were significant effects from the severity of disturbance, ontogeny, and their interactions on the number of VR modules of *H. brevisubulatum* at different times after application of treatment, especially in the experiments assessing grazing severity effects (Table 1). The number of buds significantly increased under different grazing severities at the jointing stage and under light and medium severities at the flowering stage at 1 week after recovery growth (*P* < 0.05) (Figure 1A1), under medium severity at the jointing stage and under light and medium severities at the flowering stage at 3 weeks after recovery growth (Figure 1A2), and under light severity at the jointing stage at 7 weeks after recovery growth (Figure 1A3). The number of buds significantly decreased under heavy severity at the flowering stage at 7 weeks after recovery growth (Figure 1A3). Medium severity at the jointing stage at 1 and 3 weeks after recovery growth significantly increased the number of juvenile tillers (Figures 1B1,B2), while different severities at the jointing stage significantly decreased this number at 7 weeks after recovery growth (Figure 1B3). There was no significant difference among the different severities at the flowering stage at 1, 3, and 7 weeks after recovery growth (Figures 1B1–B3). The number of tillers significantly decreased under different severities at the jointing and flowering stages at 1 and 3 weeks after recovery growth (Figures 1C1,C2). There was no significant difference in the number of tillers among the different severities at the jointing stage, while different severities at the flowering stage significantly decreased at 7 weeks after recovery growth (Figure 1C3). In examining the differences between different stages at the same grazing severity, it was found that the number of buds at the jointing stage under different severities at 1 week after recovery growth (Figure 1A1) and under light and heavy severities at 7 weeks after recovery growth (Figure 1A3), the number of juvenile tillers under medium severity at 3 weeks after recovery growth (Figure 1B2) and different severities 7 weeks after recovery growth (Figure 1B3), and the number of tillers under light and medium severities at 7 weeks after recovery growth (Figure 1C3) were greater than those at the flowering stage. These results indicated that simulated grazing by herbivores at different stages of growth first promoted the VR of *H. brevisubulatum* tufts and produced more buds, and this effect at the jointing stage was greater than that at the flowering stage. With the prolongation of recovery growth time, the harmful effect of different grazing severities on the sprouting and formation of tillers gradually decreased, and the numbers of tillers returned to the same levels as the control when grazed at the jointing stage at 7 weeks after recovery growth.

**TABLE 1:** Summary of two-way ANOVA analyses to assess the effects of severity of disturbance (S) and ontogeny (O) on the number of VR modules of *H. brevisubulatum* at different times after application of treatment (1, 3, and 7 weeks) (*F*-values).

**Different times after application of treatment**	**Indexes**	**S**	**O**	**S × O**
1 week	Bud	14.13**	45.52**	4.95**
	Juvenile tiller	1.95 ns	4.76*	0.08ns
	Tiller	266.54**	0.82 ns	0.57ns
3 weeks	Bud	17.69**	0.18 ns	3.94*
	Juvenile tiller	6.93**	12.61**	4.60**
	Tiller	99.26**	0.90 ns	4.34*
7 weeks	Bud	5.55**	34.26**	2.33ns
	Juvenile tiller	3.55*	45.99**	1.30ns
	Tiller	6.51**	18.61**	6.19**

ns, *, and ** indicate that there was no significant difference (p > 0.05), a significant difference (p < 0.05), and an extremely significant difference (p < 0.01) of different factors and their interaction with the number of VR modules of H. brevisubulatum, respectively.

### The Composition of Vegetative Reproduction Modules

**FIGURE 2:**
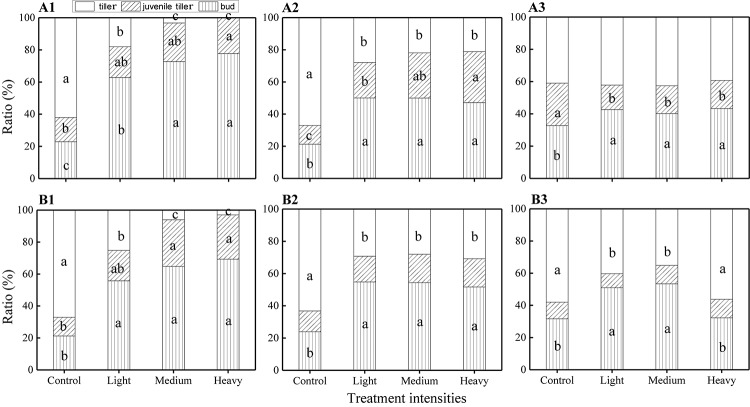
Comparison of the ratio of VR modules among different grazing severities and at different growing stages of *H. brevisubulatum* tufts at three periods of recovery growth. **A1–A3** represent the ratio of VR modules when the grazing treatment that occurred at the jointing stage at 1, 3 and 7 weeks after recovery growth, respectively. **B1–B3** represent the ratio of VR modules when the grazing treatment that occurred at the flowering stage at 1, 3 and 7 weeks after recovery growth, respectively. Different lowercase letters represent significant differences among different clipping severities (*p* ≤ 0.05).

The ANOVA test results showed that there were significant effects from the severity of disturbance, ontogeny, and their interactions on the composition of VR modules of *H. brevisubulatum* at different times after application of treatment (Table 2). The unclipping treatment at both stages showed that the highest ratio was found in the tiller, followed by the bud and juvenile tiller. Different grazing severities at both stages showed that the highest ratio was found for the bud (Figure 2). Compared with the unclipping treatment, different severities at the jointing and flowering stages significantly increased the ratio of the buds and decreased the ratio of tillers (Figure 2). Different severities at the jointing stage at 1 and 3 weeks after recovery growth increased the ratio of juvenile tillers (Figures 2A1,A2) and significantly decreased the ratio at 7 weeks after recovery growth (Figure 2A3). Medium and heavy severities at the flowering stage at 1 week after recovery growth significantly increased the ratio of juvenile tillers (Figure 2B1), while there was no significant difference among the different severities at 3 and 7 weeks after recovery growth (Figures 2B2,B3). The ratio of different VR modules increased or decreased most at 1 week after recovery growth under different grazing severities at both stages, and the increasing or decreasing trend gradually weakened with the prolongation of the recovery growth time (Figure 2). These results indicated that grazing changed the life history pattern of VR modules and mainly increased the ratio of buds and decreased the ratio of juvenile tillers. The difference in the composition of VR modules among different grazing severities gradually decreased with the prolongation of recovery growth time.

**TABLE 2:** Summary of the results of two-way ANOVA analyses of the effects of severity of disturbance (S) and ontogeny (O) on the composition of VR modules of *H. brevisubulatum* at different times after application of treatment (1, 3, and 7 weeks) (*F*-values).

**Different times after application of treatment**	**Indexes**	**S**	**O**	**S × O**
1 week	Bud	59.84**	4.71*	0.27ns
	Juvenile tiller	5.74**	0.54 ns	0.73ns
	Tiller	302.44**	7.76**	0.44ns
3 weeks	Bud	31.36**	4.29*	0.24ns
	Juvenile tiller	6.41**	12.46**	2.37ns
	Tiller	74.23**	1.29 ns	1.18ns
7 weeks	Bud	17.97**	1.10 ns	10.27**
	Juvenile tiller	2.58 ns	28.07**	2.14ns
	Tiller	8.33**	12.81**	12.74**

ns, *, and ** represent that there was no significant difference (p > 0.05), a significant difference (p < 0.05), and an extremely significant difference (p < 0.01) in different factors and their interaction on the composition of VR modules of H. brevisubulatum, respectively.

### Compensation Index of Vegetative Reproduction

**FIGURE 3:**
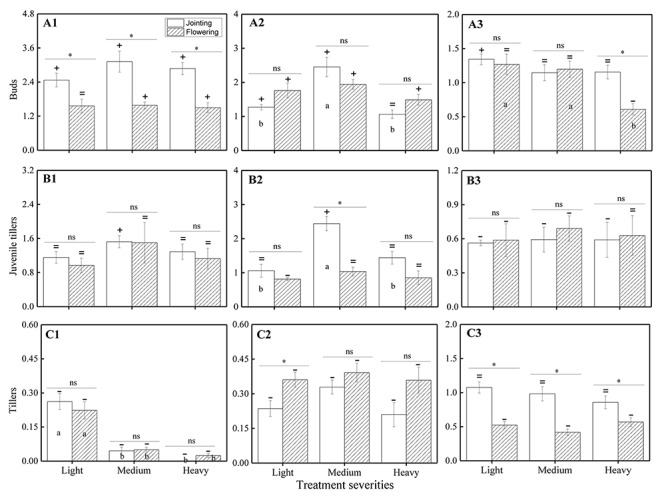
Comparison of the CI based on the number of VR modules among different grazing severities and at different growing stages of *H. brevisubulatum* tufts at three periods of recovery growth. **A1–A3** represent the CI of buds at 1, 3 and 7 weeks after recovery growth, respectively. **B1–B3** represent the CI of juvenile tillers at 1, 3 and 7 weeks after recovery growth, respectively. **C1–C3** represent the CI of tillers at 1, 3 and 7 weeks after recovery growth, respectively. Different lowercase letters represent significant differences among different clipping severities (*p* ≤ 0.05). * and ns indicate that there was a significant difference (*p* ≤ 0.05) and no significant difference, respectively, between the jointing stage and flowering stage.

The ANOVA test results showed that there were significant effects from the severity of disturbance, ontogeny, and their interactions on the compensation index of VR modules of *H. brevisubulatum* at different times after application of treatment (Table 3). The differences in compensation index among different grazing severities and different stages were consistent with the change in the number of VR modules. When comparing the CI with 1, the number of buds showed overcompensation under different severities at the jointing and flowering stages (except complete compensation under light severity at the flowering stage) at 1 week after recovery growth (Figure 3A1), while the number of juvenile tillers mainly showed complete compensation, except for overcompensation under medium severity at the jointing stage (Figure 3C1). The number of buds mainly showed overcompensation (Figure 3A2), the number of juvenile tillers showed complete compensation (Figures 3B1,B2), and the number of tillers showed undercompensation (Figure 3C2) under different severities at both stages at 3 weeks after recovery growth, which was similar to that at 1 week after recovery growth. At 7 weeks after recovery growth, the number of buds mainly showed complete compensation, except for overcompensation under light severity at the jointing stage and undercompensation under heavy severity at the flowering stage (Figure 3A3). The number of juvenile tillers showed undercompensation, except for complete compensation under heavy severity at the flowering stage (Figure 3B3). The number of tillers showed complete compensation under different severities at the jointing stage and showed undercompensation at the flowering stage (Figure 3C3). The above results indicated that the number of buds, juvenile tillers, and tillers after long-term recovery growth mainly showed complete compensation, undercompensation and complete compensation, respectively.

**TABLE 3:** Summary of the results of two-way ANOVA analyses of the effects of severity of disturbance (S) and ontogeny (O) on the compensation index of VR modules of *H. brevisubulatum* at different times after application of treatment (1, 3, and 7 weeks) (*F*-values).

**Different times after application of treatment**	**Indexes**	**S**	**O**	**S × O**
1 week	Bud	0.97 ns	42.80**	0.95ns
	Juvenile tiller	1.65 ns	0.36 ns	0.06ns
	Tiller	59.04**	0.02 ns	0.96ns
3 weeks	Bud	14.37**	0.85 ns	4.94*
	Juvenile tiller	11.94**	28.76**	6.12**
	Tiller	1.81 ns	10.51**	0.55ns
7 weeks	Bud	7.59**	7.36*	4.02*
	Juvenile tiller	0.13 ns	0.25 ns	0.05ns
	Tiller	0.98 ns	55.65**	2.04ns

ns, *, and ** represent that there was no significant difference (p > 0.05), a significant difference (p < 0.05), and an extremely significant difference (p < 0.01), respectively, in different factors and their interaction on the compensation index of VR modules of H. brevisubulatum.

## Discussion

### Renewal Capacity of Vegetative Reproduction and Grazing Tolerance at Different Growth Stages of Plants

The population recruitment and renewal of clonal plants occur primarily via VR (Harper, 1977; Benson and Hartnett, 2006; Sosnová et al., 2010). For perennial grasses, the tiller nodes on the earth’s surface are important for VR, and the number of buds at the tiller node can be used to measure the renewal capacity of VR (Benson et al., 2004; Ott and Hartnett, 2015b). However, the bud produced by VR will grow further and yield juvenile tiller and tiller in the growing season (Fidelis et al., 2014; Chen et al., 2016). Therefore, the number of juvenile tillers and tillers also indicates the renewal capacity of VR. Studies have shown that grazing clouds promote the sprouting of bud for clonal plants and improve the VR capacity (Dalgleish et al., 2012; Fidelis et al., 2014), and the renewal capacity of VR was stronger when grazed occurred in earlier periods (Sullivan and Howe, 2009; Gruntman and Novoplansky, 2011).

The number of buds for one tuft increased to different degrees during different recovery growth periods, and the increase at the jointing stage was generally higher than that at the flowering stage. The results indicated that the renewal capacity of VR was higher at the jointing stage than at the flowering stage. Previous studies have shown that the renewal capacity of VR was related to the grazing tolerance of clonal plants (Huhat et al., 2000; Lehtilä, 2000; Ott and Hartnett, 2012; Deng et al., 2015); therefore, the results showed that the grazing tolerance of the *H. brevisubulatum* population at the jointing stage was higher than that at the flowering stage.

The difference in the grazing tolerance of the *H. brevisubulatum* population at different growth stages may be related to the rhythm of plant growth and development. Research has shown that the responses of plants to grazing are different at different growing stages due to differences in structure, storage capacity, and physiological regulation (Boege, 2005; Boege and Marquis, 2005; Hanley and Fegan, 2007). The tufts of the *H. brevisubulatum* population at the jointing stage were mainly useful for vegetative growth. The growth of functional leaves was vigorous, and the physiological response to foraging apical tissue was relatively sensitive. Plants can produce and accumulate photosynthetic products during growth. Therefore, the numbers of tillers under different severities completely returned to the control levels at 7 weeks of recovery growth. However, most stored nutrients are used for sexual reproduction in the upper part of the tiller at the flowering stage, and the functional leaves in the bottom part of the tiller begin to senesce (Zhang et al., 2012). Then, the regulatory function of the physiological response to foraging apical tissue is weakened. Therefore, the numbers of tillers under different severities did not return to the control levels at 7 weeks of recovery growth. In summary, the VR capacity of the *H. brevisubulatum* population was stronger at the jointing stage, and grazing tolerance was higher after grazing (Oesterheld and Mcnaughton, 1991; Tucker and Avila-Sakar, 2010; Gruntman and Novoplansky, 2011). For local grassland management practices, regardless of whether the grazing severity is light or heavy, there will be no harmful effect of grazing at the jointing stage on population regeneration and the production of *H. brevisubulatum* grassland.

### Changes in the Life History Pattern of Vegetative Reproduction Composition and Recovery Capacity

The population of clonal grass consists of bud, juvenile tiller without tillering node, vegetative tiller with tillering node, and reproductive tiller with fruit at different stages of life history for VR (Yang and Zhu, 2011). In different habitats or disturbed conditions at different stages during the growing season, the transformation from bud to juvenile tiller and from juvenile tiller to tiller could change the life history pattern of VR modules of clonal grasses (Ott and Hartnett, 2015a). Considering the composition of different VR modules, the bud is the basic module for supplementation and potential population renewal. The juvenile tiller and tiller are basic modules of the population and the main modules of population matter production, and the capacity for matter production of juvenile tillers increases with the growth of the module (Yang and Zhu, 2011). The output law of tiller from bud after grazing is one of the most important mechanisms conferring plant resilience to herbivory and is conducive to recovery after grazing for damaged populations (Tiffin, 2000). The life history pattern of module composition of herbaceous populations is the basis for evaluating population dynamics and matter production (Li et al., 2012).

In our study, the highest ratio of VR module was tiller under the control treatment, while the ratio of bud was the highest under simulated grazing treatment. The results indicated that the main function of the clonal population is the production of tillers when there is no interference from herbivores. However, the main function of the clonal population is to produce more buds to ensure the potential vegetative reproduction of the population, which is important for the resistance to the interference of herbivores and the productivity recovery after the interference (Dalgleish and Hartnett, 2009; Chen et al., 2016). The change in life history pattern of vegetative propagation components after simulated grazing disturbance indicates the change in the strategies of vegetative reproduction. The tiller is the main VR module of the population with matter production (Yang and Zhu, 2011). The ratio of tillers under different grazing severities at the jointing stage at 7 weeks of growth recovery was consistent with that under the control treatment. The results indicated that the population dynamics under grazing treatments were similar to those under the control treatment, and the capacity for matter production was completely restored. However, the capacity for matter production was not fully restored at the flowering stage. Therefore, the recovery capacity of the *H. brevisubulatum* population after the interference of grazing at the jointing stage was higher than that at the flowering stage.

### Mechanism of Compensatory Tillering

According to the physiological principle of plant nutrient supply for apical dominance, apical dominance would be broken when the top of the plants is grazed by herbivores, promoting resprouting after damage and improving the vegetative propagation of clonal plants (Thomson et al., 2003; Da Silveira Pontes et al., 2010; Qian et al., 2014). The compensatory growth of plants first occurs after grazing (Zhao et al., 2008). However, due to the senescence and death of plant leaves, the continuous growth and production of VR modules are the basis for plants to achieve compensatory growth in matter production (Yuan et al., 2019). The maintenance of belowground bud banks in grassland ecosystems enables plants to survive adverse environments, such as grazing disturbance and drought (Dalgleish and Hartnett, 2009; VanderWeide and Hartnett, 2015). Therefore, the promotion of bud sprouting after grazing is important for plant compensatory growth, and the output of tiller with multi-leaves from the bud finally promotes the compensatory growth of plants (Chen et al., 2016; Nakahara et al., 2018). Therefore, the VR of the population and the turnover rate of modules after grazing disturbances, especially the turnover rate of tiller from bud, were considered the major internal mechanisms of plant compensatory growth (Strauss and Agrawal, 1999; Tiffin, 2000).

In our study, the increase in bud number and the decrease in tiller number in the *H. brevisubulatum* population after grazing gradually decreased with prolonged recovery growth time. Compared with that at 1 week after recovery growth, the number of tillers at 7 weeks after recovery growth under the control treatment at the jointing and flowering stages increased by 0.22 and 0.26 times, respectively. However, the number of tillers under light severity, medium severity, and heavy severity increased by 4.04, 25.83, and 93.67 times at the jointing stage, respectively, and increased by 2.04, 9.90, and 29.69 times at the flowering stage, respectively. These results indicate that different grazing severities and initial grazing times promoted the transformation of bud to tiller. The transformation of buds to tillers increased with increased grazing severity, and the number of buds transformed to tillers was higher at the jointing stage. Therefore, the number of tillers under different grazing severities at the jointing stage at 7 weeks after recovery growth was similar to that under the control treatment, which showed complete compensation. However, the number of tillers showed undercompensation at the flowering stage. In summary, the mechanism of compensatory vegetative propagation of plants is that grazing provoked vegetative regeneration or resprouting after damage and promoted a significant increase in the number of bud banks and the transformation among VR modules, especially promoting the turnover process of bud to tiller.

### Theoretical and Practical Significance of the Compensation Index Based on the Number of Modules

Compensatory growth is a positive response of plants to damage and is generally measured by the traits related to fitness. In fact, changes in biomass and the number of plant organs indicate the compensatory growth capacity (Mundim et al., 2012; Erbilgin et al., 2014; Scholes et al., 2016). In our study, the numbers of buds, juvenile tillers and tillers produced by VR were used to calculate the CI. According to the difference in the CI among different grazing severities and different grazing periods and the change in the CI at different recovery growth times, the CI can comprehensively effectively indicate compensatory VR under different simulated grazing treatments and provide scientific guidance for grassland management. We can determine a plan for rotational grazing and provide practical scientific guidance for grassland grazing management.

CIs of buds under different severities at the jointing stage were higher than those at the flowering stage. The results indicated that the compensatory growth of VR and recovery capacities of the *H. brevisubulatum* population after simulated grazing at the jointing stage were higher than those at the flowering stage. The number of tillers mainly showed undercompensation, while showed complete compensation at the jointing stage at 7 weeks. The results indicated that different grazing severities at both growing stages had adverse effects on the matter production of the *H. brevisubulatum* population. The adverse effects at the jointing stage were completely eliminated at 7 weeks after recovery growth with the prolongation of recovery growth time, and the biomass production showed complete compensation or overcompensation (unpublished data). However, the adverse effects at the flowering stage continued. Therefore, the grazing rotation of grassland when the productivity was completely restored was 50 days at the jointing stage and longer at the flowering stage. The duration for which the adverse effects of different grazing severities at the flowering stage would be eliminated and whether they could be eliminated in this growing season need further study.

## Conclusion

In this study, the clonal tussock grass *Hordeum brevisubulatum* was selected to study the effect of simulated grazing. Four clipping severities at the jointing and flowering stages were implemented, and three sampling times were used at different recovery durations. We found that simulated grazing increased the number of buds. The promotion was greater in the jointing stage than the flowering stage, and the increase decreased with prolonged recovery growth time. The number of tillers significantly decreased, and the decreasing effect weakened with prolonged recovery growth time. The tiller number showed undercompensation at 1 and 3 weeks after recovery growth, while it showed complete compensation at the jointing stage and undercompensation at the flowering stage at 7 weeks after recovery growth. Our results suggest that different simulated grazing severities at the jointing stage could increase the output of tiller from bud and improve the compensatory growth capacity.

## Data Availability Statement

The datasets generated for this study are available on request to the corresponding author.

## Author Contributions

YY and JY designed the experiments. JY performed the experiments. JY and HL analyzed the data and wrote the manuscript. All authors read and approved the manuscript.

## Conflict of Interest

The authors declare that the research was conducted in the absence of any commercial or financial relationships that could be construed as a potential conflict of interest.
